# Small cell carcinoma in a bladder diverticulum: a rare case report and literature review

**DOI:** 10.3389/fonc.2026.1837985

**Published:** 2026-06-04

**Authors:** Zhengping Yang, Jie Zhang, Guantian Yang, Zheng Zeng, Xing Jin

**Affiliations:** 1 Department of Urology, Jiangsu Province Hospital on Integration of Chinese and Western Medicine; Jiangsu Province Academy of Traditional Chinese Medicine, Nanjing, China; 2 Department of Oncology, Nanjing Pukou District Hospital of Traditional Chinese Medicine, Nanjing, China; 3 Department of Pathology, Jiangsu Province Hospital on Integration of Chinese and Western Medicine; Jiangsu Province Academy of Traditional Chinese Medicine, Nanjing, China

**Keywords:** bladder diverticulum, clinicopathological features, diagnostic, small cell carcinoma, treatment

## Abstract

**Background:**

Small cell carcinomas arising from bladder diverticula are extremely rare. The highly malignant nature of small cell carcinoma, combined with the absence of a muscularis propria in the diverticular wall, makes these tumors prone to early invasion and metastasis, resulting in an extremely poor prognosis.

**Case presentation:**

We report a rare case of small cell carcinoma occurring in a bladder diverticulum. An 80-year-old woman presented to the hospital with gross hematuria. Computed tomography urography and cystoscopy confirmed the presence of tumors within the bladder diverticulum. Partial cystectomy was performed. Histopathological examination of the specimen revealed small cell carcinoma, and perivesical fat tissue invasion and intravascular invasion were detected. Radical cystectomy, adjuvant chemotherapy, and radiotherapy were refused. Later, tumor recurrence occurred, and the patient died 8 months after surgery.

**Conclusion:**

Small cell carcinoma of the bladder diverticulum represents an extremely rare and aggressive malignancy, with only a handful of cases reported in the medical literature. This scarcity poses significant diagnostic and therapeutic challenges and underscores the need for increased awareness and further research to improve understanding and management of this complex condition.

## Introduction

Primary tumors occurring in urinary bladder diverticula are exceedingly rare, with a reported incidence ranging from 0.8% to 10.0% (1). Among them, the most common histologic type is urothelial carcinoma, followed by rare occurrences of squamous cell carcinoma, small cell carcinoma, adenocarcinoma, and sarcoma. Small cell carcinoma arising from a bladder diverticulum is extremely rare. According to searches of PubMed and Web of Science, fewer than 10 cases have been reported to date. It is an aggressive neoplasm characterized by high rates of metastases, more aggressive behavior, and poorer prognosis. We report a new case of this rare tumor and review the relevant literature.

## Case presentation

An 80-year-old woman presented with painless, intermittent gross hematuria of 3 months’ story duration. The patient had hypertension but did not have any other comorbidities or a history of nicotine use. Laboratory results revealed numerous erythrocytes in the urine. Ultrasound examination of the urinary system revealed a hypoechoic area protruding outward from the right bladder wall. Noncontrast computed tomography and computed tomography urography revealed a diverticulum located on the right posterior bladder wall, containing irregular solid lesions (**Figure 1**). Local invasion or metastasis to lymph nodes or distant organs was not observed on CT images. Cystoscopy confirmed the presence of a diverticulum on the right bladder wall, containing two papillary neoplasms, which are typical features of urothelial carcinoma, one of which exhibited surface calcification (**Figure 2**). Moreover, urothelial carcinoma has been reported as the most common pathological type of tumors within bladder diverticula; therefore, urothelial carcinoma was suspected. Furthermore, preoperative imaging studies revealed no obvious evidence of local invasion or distant metastasis. These considerations led us to believe that performing a partial cystectomy directly would achieve complete tumor resection. As a result, a preoperative biopsy was omitted, and a partial cystectomy was performed, with the tumor excised along a 2-cm circumferential margin.

**Figure 1:**
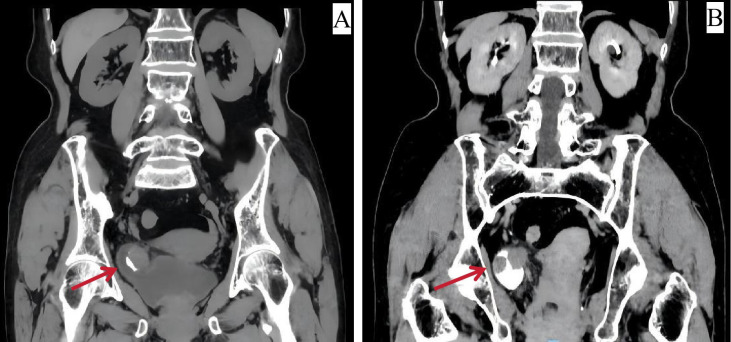
Noncontrast computed tomography revealed a solid mass with a calcified surface in a bladder diverticulum located on the right posterior bladder wall [(**A**), red arrow]. Computed tomography urography revealed two masses in the diverticulum [(**B**), red arrow].

**Figure 2:**
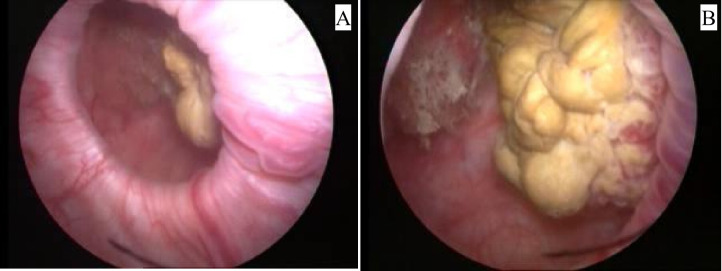
Cystoscopy revealed two tumorous masses in the bladder diverticulum **(A)**, one of them with a calcified surface **(B)**.

Postoperative pathological examination revealed sheets and nests of a uniform population of small round cells (**Figure 3A**). Focal transitional cell carcinoma was seen (**Figure 3B**). Tumor invasion was also observed in blood vessels. Although the specimen margins were negative, cancer tissue was observed throughout the entire bladder wall, extending into the perivesical fat tissue.

**Figure 3:**
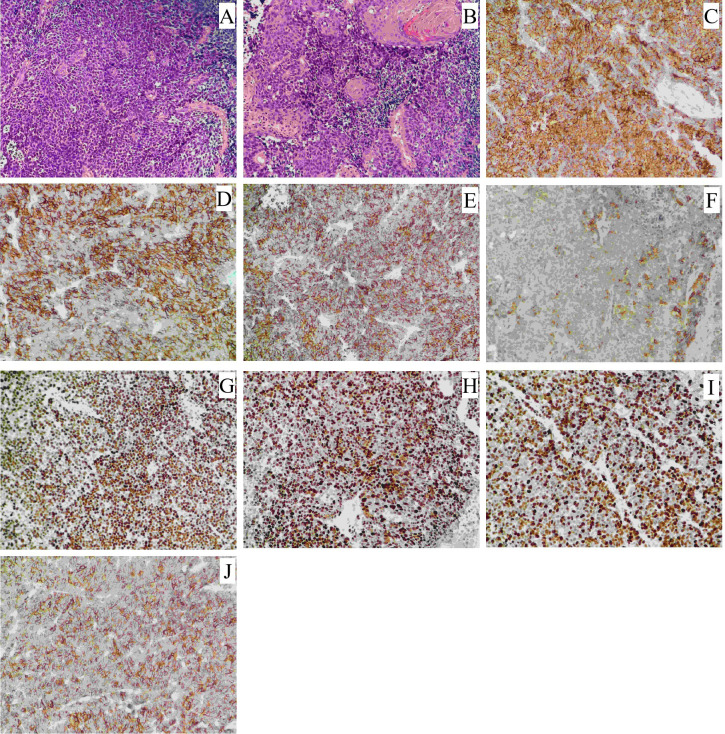
Hematoxylin and eosin (H&E) staining showed sheets and nests of a uniform population of small round cells [**(A)**, × 200]. Focal transitional cell carcinoma was observed [**(B)**, × 200]. Immunohistochemical staining showed positivity for CKpan [**(C)**, × 200], CD56 [**(D)**, × 200], CK7 [**(E)**, × 200], EMA [**(F)**, × 200], P63 [**(G)**, × 200], P53 [**(H)**, × 200], Ki-67 [**(I)**, × 200], and CD117 [**(J)**, × 200].

Immunohistochemistry revealed positivity for pan cytokeratin (CKpan), cluster of differentiation 56 (CD56), cytokeratin 7 (CK7), epithelial membrane antigen (EMA), protein 63 (P63), protein 53 (P53), Kiel-67 antigen (Ki-67) (90%+), and cluster of differentiation 117 (CD117) (**Figures 3C–J**). Positive CKpan and epithelial membrane antigen (EMA) strongly suggested that the lesion originated from urothelial tissue. Positivity for P53 and a Kiel-67 antigen (Ki-67) proliferation index of up to 90% indicated the malignant nature of the lesion. The positive protein (P53) positivity was particularly notable, as it fits the characteristics of small cell carcinoma with high proliferative activity. P63 positivity supported the diagnosis of urothelial carcinoma. Positive CD56 and CD117 indicated neuroendocrine differentiation. CD117 is a tyrosine kinase receptor that activates multiple signaling pathways promoting tumor cell proliferation, survival, and migration. Therefore, its expression may indicate more aggressive biological behavior of the tumor rather than merely serving as a marker of neuroendocrine differentiation. CK7 is an intermediate filament protein found in most glandular and transitional epithelia. Its positivity is typically seen in small cell carcinoma. Based on the histological and immunohistochemical findings, the final diagnosis was primary small cell carcinoma with components of urothelial carcinoma. The pathological stage was pT3NxMx according to the American Joint Committee on Cancer (AJCC) staging criteria. Chemotherapy was recommended but, unfortunately, was refused by the patient.

Five months after surgery, the patient presented again with gross hematuria and worsening pelvic pain. A CT scan of the abdomen and pelvis revealed a mass in the right pelvic cavity, involving the right bladder wall and right pelvic lymph nodes. Recurrence and progression of the cancer were considered. Given the patient’s age and performance status, she refused radical cystectomy, chemotherapy, and radiotherapy after being informed that such treatments might be ineffective against small cell variants and could cause intolerable side effects. She agreed only to receive palliative therapy. The patient died 8 months later after being diagnosed with metastases to the bone and liver.

## Discussion

Small cell carcinoma occurring within a bladder diverticulum has rarely been reported. According to a search of PubMed and Web of Science, the first case was reported in 2007, and only six cases have been reported to date (**Table 1**).

**Table 1:** Summary of small cell carcinoma in a bladder diverticulum in five cases and in a urethral diverticulum in one case.

Author, year	Age	Sex	Stage	Histological variants	Treatment	Outcomes
Tamura, 2007 (2)	64	Male	NR	UC	PC and BCG IVT	LFU at 16 months post-op
Labanaris, 2009 (3)	65	Male	cT3N0M0	NR	PC with LND, EPC, RT	Dead at 16 months post-op
Dong, 2013 (4)	50	Male	cT4N0M0	UC	RC with LND, EPC, and RT	Dead at 29 months post-op
Tudor, 2014 (5)	66	Male	NR	NR	TUR	NR
Nagai, 2014 (6)	73	Female	pT3aN0M0	UC	PC and GCC	Dead at 12 months post-op
Takizawa, 2019 (7)	80	Male	cT3bN0M0	NR	Neoadjuvant IPC and PC	LFU at 14 months post-op

NR, not reported; UC, urothelial carcinoma; PC, partial cystectomy; BCG, Bacillus Calmette–Guérin; IVT, intravesical therapy; LND, lymph node dissection; EPC, cisplatin and etoposide chemotherapy; RT, radiotherapy; RC, radical cystectomy; TUR, transurethral resection; GCC, gemcitabine and cisplatin chemotherapy; IPC, cisplatin and irinotecan chemotherapy; LFU, lost to follow-up; post-op, after surgery.

Urinary bladder diverticula are abnormal bulges that project outward from the bladder wall. They can be of any size, ranging between less than 1 cm and more than 10 cm, with an average size of around 5 cm (8, 9). Bladder diverticula are classified as congenital or acquired. Congenital diverticula are most common in boys under the age of 10 and are considered true diverticula because they contain all layers constituting the main bladder. They also tend to be solitary and are not usually associated with bladder trabeculations (10). As opposed to congenital diverticula, bladder diverticula in adults are referred to as acquired diverticula or pseudodiverticula because of the absence of a muscle layer and are mostly found in persons with bladder outlet obstruction or neurogenic bladder (11, 12). Increased intravesical pressure can result in herniation of the mucosa and submucosa, leading to the formation of a pseudodiverticulum. The walls of pseudodiverticula, which lack muscle fibers, tend to be dysfunctional and disordered, causing poor bladder emptying and urinary stasis. These conditions can increase the risk of urinary tract infection, bladder calculi, and neoplasms because of prolonged exposure to carcinogens such as aromatic amines found in cigarette smoke (9, 10, 13–15). According to Barnier’s study, among 611 patients treated for bladder tumors at a single medical center, eight cases of primary carcinoma originating from diverticula were identified, accounting for 1.3% of all bladder tumors (13). The most prevalent histological type of malignant tumor in diverticula is urothelial carcinoma, followed by squamous cell carcinoma (8–10, 12). As indicated by Voskuilen (16), histological variants are found in 18% of diverticular tumors, with squamous differentiation and sarcomatoid differentiation being the most frequent. The variant of small cell carcinoma originating in bladder diverticula is very rare. To date, fewer than 10 cases have been reported, making it one of the rarest tumors of the bladder.

The clinical symptoms of bladder diverticulum tumors are not different from those of other bladder tumors. The most common symptom is painless gross hematuria, followed by urinary retention, urinary tract infection, and dysuria (13, 17). The initial evaluation of suspected tumors in bladder diverticula is conducted following a similar series of tests to those employed for the assessment of tumors in the main bladder, including imaging, cytological examination, cystoscopy, and histopathological examination. Computed tomography urography (CTU) is the most accurate imaging modality for detecting and staging tumors in the bladder diverticulum. A retrospective case series including 34 patients with tumors in bladder diverticula showed that, in such patients, the sensitivity for detecting tumors in diverticula and their extravesical extension reached 94% and 86%, respectively (18). In patients who are contraindicated for CTU and cystoscopy, or when a mass is suspected in a bladder diverticulum but cannot be confirmed through ultrasonography and CTU, MRI is required to identify the tumor origin (19). Although high sensitivity in urothelial carcinoma has been reported for high-grade tumors and carcinoma *in situ*, cytology is recommended only as an auxiliary diagnostic method for detecting tumors in bladder diverticula and for the follow-up assessment of patients who have undergone bladder-conserving treatment for tumors in bladder diverticula due to its lower sensitivity for low-grade tumors (19, 20). Regardless of whether imaging or cytological examinations can definitively confirm or rule out a diagnosis of tumors in bladder diverticula, diagnostic cystoscopy is mandatory unless contraindications to cystoscopy exist (21). Routine cystoscopy to assess lesions in a bladder diverticulum can be difficult, as the neck of the diverticulum may be too narrow or the angle of entry may be too acute for the cystoscope to access the diverticulum. In such cases, a different viewing-angle telescope lens or a flexible ureteroscope may be of value. Cystoscopic biopsy should also be performed if anatomy permits. It is unclear whether diagnostic bladder biopsy increases intravesical recurrence after bladder-preserving surgery for bladder tumors. However, performing diagnostic ureteroscopy before surgery or performing a biopsy during diagnostic ureteroscopy has been identified as a risk factor for intravesical recurrence (22). The patient reported in this case presented with the typical symptom of hematuria and typical CTU imaging features of a bladder diverticulum tumor. Cystoscopy also revealed the typical morphology of a bladder tumor with calcification. Therefore, no further biopsy was attempted to assess the possibility of malignancy; instead, a partial cystectomy was performed directly. Postoperative histopathology confirmed a malignancy in the bladder diverticulum. However, it was not the common urothelial carcinoma but rather small cell carcinoma, coexisting with components of urothelial and neuroendocrine carcinoma.

Small cell carcinoma arising in a bladder diverticulum is an extremely rare malignancy, and fewer than 10 cases have been reported. According to current reports, no patients with small cell carcinoma in bladder diverticula have survived for more than 3 years. The poor prognosis may be partly attributed to the deficiency of muscle fibers in the diverticulum, which facilitates early metastasis, and partly related to the highly invasive nature of small cell carcinoma (3). At present, there is no standard treatment for small cell carcinoma within bladder diverticula. In a pooled analysis of 498 cases covering all histopathological types within bladder diverticula, rather than specifically targeting a particular histopathological type, there is consensus among all reporters that tumors within bladder diverticula should be excised when diagnosed, with radical cystectomy (34.34%) as the main surgical approach, followed by partial cystectomy (26.66%) (23). One retrospective study including the largest series of urothelial carcinoma arising in bladder diverticula published so far revealed no differences in overall survival and metastasis-free survival after radical cystectomy and partial cystectomy (16). Similar results were presented in three additional studies (8, 17, 24). These findings suggest that partial cystectomy may represent a feasible surgical alternative to radical cystectomy in patients with bladder diverticulum tumors. However, these findings are largely based on urothelial carcinoma and may not apply to the more systemic nature of small cell variants. Diverticulectomy or transurethral resection may also be considered in strictly selected patients with low-grade, low-volume tumors. Transurethral resection can be technically challenging, as it may not be possible to access the diverticulum owing to a narrow neck or an acute angle of entry, as well as the surgeon’s hesitation to perform deep resections due to the potential risk of bladder perforation and tumor dissemination (19).

Due to the aggressive nature of small cell carcinoma and the tendency for early invasion, surgical treatment alone is insufficient for curative treatment, and additional therapies are typically required. Moreover, due to the rarity of this condition, evidence-based treatment guidelines are limited, and management decisions are often based on case reports and extrapolation from protocols used for small cell lung cancer or bladder urothelial carcinoma, given the histological similarities between these malignancies. The combination of cisplatin and irinotecan (IP regimen) has been successfully used in a case as neoadjuvant chemotherapy for small cell carcinoma arising in a bladder diverticulum and resulted in significant tumor shrinkage, allowing for partial cystectomy instead of more radical surgery (7). The patient remained free of recurrence at 14 months postoperatively, demonstrating the potential effectiveness of this regimen. Alternative chemotherapy protocols used as adjuvant treatment include the combination of gemcitabine and cisplatin (GC regimen) and the combination of cisplatin and etoposide (EP regimen). GC regimen is commonly used for urothelial carcinoma of the bladder and may be particularly appropriate for cases with mixed histology containing both small cell carcinoma and urothelial carcinoma components (6). EP therapy has shown activity in small cell lung cancer and may be considered an alternative treatment for small cell carcinoma in the bladder (3, 4). For patients with mixed histology tumors containing both small cell carcinoma and urothelial carcinoma, Bacillus Calmette–Guérin (BCG) intravesical therapy has been used as an adjunctive treatment following bladder-preserving surgical resection (2).

## Conclusion

Bladder carcinoma arising in a diverticulum is a rare condition that presents distinct challenges in treatment. Aggressive histology should be suspected when imaging reveals a solid mass within a diverticulum, necessitating a more urgent diagnostic biopsy. Given the rarity of small cell carcinoma of bladder diverticulum, the available literature consists primarily of case reports. Analyzing these individual cases provides valuable insights into the effectiveness of various treatment approaches and helps identify patterns that may guide future management of this challenging condition. The treatment of small cell carcinoma of the bladder diverticulum requires a multimodal approach combining surgery with neoadjuvant and adjuvant chemotherapy. The role of radiotherapy, as well as novel therapeutic agents, including targeted therapies and immunotherapies, should be explored in the treatment of small cell carcinoma of the bladder diverticulum.

## Data Availability

The original contributions presented in the study are included in the article/supplementary material. Further inquiries can be directed to the corresponding author.
